# Social inequalities and prevalence of depressive symptoms: a cross-sectional study of women in a Mexican border city, 2014

**DOI:** 10.26633/RPSP.2020.9

**Published:** 2020-02-05

**Authors:** Alheli Calderon-Villarreal, Oscar J. Mujica, Ietza Bojorquez

**Affiliations:** 1 El Colegio de la Frontera Norte El Colegio de la Frontera Norte Tijuana Mexico El Colegio de la Frontera Norte, Tijuana, Mexico.; 2 Pan American Health Organization/World Health Organization Pan American Health Organization/World Health Organization Washington, DC United States of America Pan American Health Organization/World Health Organization, Washington, DC, United States of America.; 3 Department of Population Studies El Colegio de la Frontera Norte Tijuana Mexico Department of Population Studies, El Colegio de la Frontera Norte, Tijuana, Mexico.

**Keywords:** Depression, social determinants of health, health status disparities, women’s health, border health, Mexico, Depresión, determinantes sociales de la salud, disparidades en el estado de salud, salud de la mujer, salud fronteriza, México, Depressão, determinantes sociais da saúde, disparidades nos níveis de saúde, saúde da mulher, saúde na fronteira, México

## Abstract

**Objective.:**

To assess the association between intersectional disadvantage and clinically significant depressive symptoms (CSDS), describing the magnitude of social inequalities in the prevalence of symptoms among adult women in Tijuana, Mexico.

**Methods.:**

This was a cross-sectional study. CSDS were assessed using the Centers for Epidemiological Studies Depression Scale among a probability sample of 2 345 women from 18 – 65 years of age in 2014. CSDS prevalence was calculated according to categories of three social stratifiers: socioeconomic status (SES), educational attainment, and fertility (number of children). Social inequality was measured with the slope index of inequality (SII) and the concentration index (CIx). Intersectionality among stratifiers was explored descriptively and with multivariable regression analysis.

**Results.:**

CSDS prevalence was 17.7% (95%CI: 15.1% – 21.0%). The SII and CIx showed inequity in all social stratifiers. The absolute difference in CSDS prevalence between the lowest and highest ends of the SES gradient was 21.9% (95%CI: 21.5% – 22.4%). Among the most disadvantaged women, i.e., those at the intersection of lowest SES, lowest educational attainment, and highest fertility, the CSDS prevalence was 39.5% (95% CI: 26.0% – 52.9%).

**Conclusions.:**

Disadvantage along multiple axes was associated with CSDS. Efforts to improve the mental health of women should include equity-oriented policies that address its social determinants.

## RESUMEN

**Objetivo.:**

Evaluar la asociación entre la desventaja interseccional y los síntomas depresivos clínicamente significativos (SDCS), y describir la magnitud de las desigualdades sociales en la prevalencia de síntomas en mujeres adultas de Tijuana, México.

**Métodos.:**

Se realizó un estudio transversal. Se evaluaron los SDCS utilizando la Escala de Depresión de los Centros de Estudios Epidemiológicos, en una muestra probabilística de 2 345 mujeres de 18 a 65 años, en 2014. Se calculó la prevalencia de SDCS según las categorías de tres estratificadores sociales: nivel socioeconómico, nivel educativo y fertilidad (número de hijos). Se midió la desigualdad social mediante el índice de desigualdad de la pendiente y el índice de concentración. Se exploró la interseccionalidad entre los estratificadores de manera descriptiva y con análisis de regresión multivariable.

**Resultados.:**

La prevalencia de SDCS fue de 17,7% (IC 95%: 15,1% – 21,0%). El índice de desigualdad de la pendiente y el índice de concentración mostraron desigualdad en todos los estratificadores sociales. La diferencia absoluta en la prevalencia de SDCS entre los extremos inferior y superior del gradiente de nivel socioeconómico fue de 21,9% (IC 95%: 21,5% – 22,4%). Entre las mujeres más desfavorecidas (las que se encuentran en la intersección del nivel socioeconómico más bajo, el nivel educativo más bajo y la fertilidad más alta) la prevalencia de SDCS fue de 39,5% (IC 95%: 26,0% – 52,9%).

**Conclusiones.:**

La desventaja a lo largo de múltiples ejes se asoció con SDCS. Los esfuerzos para mejorar la salud mental de las mujeres deben incluir políticas orientadas a la equidad que aborden sus determinantes sociales.

## RESUMO

**Objetivo.:**

Avaliar a associação entre a sobreposição de desvantagens e sintomas depressivos clinicamente significativos (SDCS), descrevendo a magnitude das desigualdades sociais na prevalência de sintomas entre mulheres adultas em Tijuana, México.

**Métodos.:**

Foi realizado um estudo transversal. Os SDCS foram avaliados em 2014 por meio da escala *Center for Epidemiologic Studies – Depression* (CES-D) em uma amostra probabilística de 2 345 mulheres de 18 a 65 anos. A prevalência de SDCS foi calculada de acordo com as categorias de três estratificadores sociais: nível socioeconômico (NSE), nível educacional e fertilidade (número de filhos). A desigualdade social foi medida pelo índice angular de desigualdade e pelo índice de concentração. A sobreposição entre os estratificadores foi explorada de forma descritiva e por análise de regressão multivariada.

**Resultados.:**

A prevalência de SDCS foi de 17,7% (IC95%: 15,1% a 21,0%). O índice angular de desigualdade e o índice de concentração mostraram desigualdade em todos os estratificadores sociais. A diferença absoluta na prevalência de SDCS entre os extremos mais baixo e mais alto do gradiente de NSE foi de 21,9% (IC95%: 21,5% a 22,4%). Entre as mulheres mais desfavorecidas, ou seja, as que se encontram na intersecção entre a NSE mais baixa, o menor nível educacional e a maior fertilidade, a prevalência de SDCS foi de 39,5% (IC95%: 26,0% a 52,9%).

**Conclusões.:**

A desvantagem ao longo de múltiplos eixos foi associada aos SDCS. As iniciativas para melhorar a saúde mental das mulheres devem incluir políticas orientadas para a equidade que considerem os determinantes sociais da saúde mental.

Non-communicable diseases, including mental illnesses, are a major component of the global burden of disease. According to the World Health Organization (WHO), the global prevalence of depression in 2015 was 4.4%, representing 322 million people and an 18.4% increase since 2005 (1). According to the 2017 Global Burden of Disease Study (2), depressive episodes are the 3rd leading cause of Years Lived with Disability in women (692 per 100 000) and 5^th^ in men (438 per 100 000).

WHO defines depression as a serious, yet common, medical condition that interferes with daily life (the ability to work, sleep, study, eat, and enjoy life), caused by a combination of genetic, biologic, environmental, and psychological factors (3). Depressive episodes, according to the Diagnostic and Statistical Manual of Mental Disorders—5^th^ Edition (DSM-5), are defined by symptoms such as sadness, loss of interest or pleasure, feelings of guilt or low self-esteem, changes in sleep or appetite, fatigue, and lack of concentration, which cause significant malaise or deterioration in social, work, or other areas of functioning (4). Even if the depressive symptoms do not meet the full clinical criteria for a depressive disorder or episode, they may have important social and personal consequences, and therefore require attention (5).

Depression is the result of multiple biological factors, including genetic, endocrine (6 – 9), and social factors which, when combined, can put some individuals at increased risk. Without denying the importance of biological factors, this article follows the social determinants of health (SDH) framework (10) by focusing on social aspects that may explain why certain social groups (as opposed to individuals) experience a higher and unfair burden of depression (11).

The SDH are defined as “the conditions in which people are born, grow, live, work, and age” (10). They include the differential distribution of economic, political, and other resources that result in health inequities (10, 12). The WHO Commission on the SDH has determined that in all countries, health and illness follow a social gradient in which lower social position is generally associated with worse health (10). Given that Latin America has the highest socioeconomic inequalities in the world and considering the growing importance of mental illnesses, a comprehensive study of the relationship between social stratifiers and mental health across and within Latin American nations was essential (13).

Gender is a major social determinant of health. All over the world, women and girls experience social disadvantages that negatively impact their health (10). The prevalence of depression is higher among women than it is among men (1), a disparity reported in diverse regions, cultures, and social contexts (14). While depression in women might be partly due to biological factors (6, 7, 9), gender inequities regarding power and access to resources and the differential valuation of female identity likely explain the depression gender-gap (15). However, not all women are equally disadvantaged. As the theory of intersectionality proposes, multiple overlapping vulnerabilities interact to place certain social groups at a greater disadvantage (16, 17).

The literature on the social determinants of mental health shows that the worst mental health outcomes are concentrated in population subgroups with lower socioeconomic status (SES), income, and educational attainment (13, 18 – 20). Disadvantages in these social stratifiers affect life conditions, and often, result in illness and precarious health status, including higher risk of depressive symptoms (13, 21). High fertility may also raise the risk of depression, especially after the 5^th^ child. This increased risk is possibly due to increased economic burden and its associated stressors, the caregiving workload, and/ or the social isolation of mothers (22). SES, educational attainment, and the number of children are social stratifiers. Some women may be at the intersection of two or more disadvantaged positions in the social space defined by those stratifiers.

Prompted by the paucity of research on this topic in Latin America, this study sought to understand the social distribution of depressive symptoms, as well as the combined effect of multiple axes of disadvantage and the magnitude of any differential distribution of depressive symptoms among social groups. An apt place to explore associations between social stratifiers and depressive symptoms is Tijuana, Mexico—a city of over 1.6 million inhabitants (23) that shares a border with San Diego County, United States, and exhibits social, economic, and cultural contrasts among diverse groups, including both internal and international migrants. To our knowledge, only one previous study, conducted over three decades ago, has investigated the prevalence of depression in a representative sample of the city’s population (24). That study, which employed an older version of the same instrument applied in the present one, found that clinically significant depressive symptoms (CSDS) had a prevalence of 33% among women and an association with low socioeconomic status.

The objective of this study was to assess the association between intersectional disadvantage and CSDS, describing the magnitude of social inequalities in the prevalence of these symptoms among adult women in Tijuana, Mexico.

## MATERIALS AND METHODS

This was a cross-sectional study of a probability sample of 2 345 female residents, 18 – 64 years of age, in Tijuana, Mexico, conducted in 2014. The study employed a multistage sampling design, beginning with the selection of Basic Geographic Statistical Areas (AGEB) in strata defined by degree of marginalization (low/medium/high), provided by the Mexican National Population Council (25). City blocks were selected in each AGEB. All homes on each selected block were visited. In each home, one woman who met the inclusion criteria (18 – 65 years of age and providing informed consent) was selected and she completed an oral survey. If multiple eligible women were present, the respondent was randomly selected.

### Depressive symptoms scale

The Center for Epidemiological Studies Depression Scale (CES-D) 10-item version (26, 27), validated for use in Mexican populations (5), was administered to assess the presence of CSDS during the prior week. Each participant was asked how often during the past week she had experienced the following symptoms: Were you bothered by things that don’t normally bother you? Did you have trouble paying attention? Did you feel depressed? Did you feel that everything you did required effort? Did you feel optimistic? Did you feel fear? Did you have trouble sleeping? Were you happy? Did you feel lonely? Did you have no desire to do anything? Response options for each item ranged on a Likert scale from “rarely or none of the time” (0 points) to “all of the time” (3 points). Questions with inverse values were re-coded. Of a total possible score of 30 points, a value of ≥ 10 was considered to be the threshold for a binary index of presence of depressive symptoms.

### Social stratifiers

The prevalence of CSDS was compared among levels of three social stratifiers: SES, educational attainment, and fertility (number of children). SES was estimated according to household resources, with a methodology based on the recommendations of the Demographic and Health Surveys (28). The index captures the presence of household assets, such as: a bathroom in the home, a gas/electric stove, electricity, boiler/water heater, refrigerator, washer, telephone landline, cell phone, television, satellite/cable television, computer, Internet access, a domestic worker who visits some days per week, a daily domestic worker, and a private automobile. These were combined in an index using principal components analysis to obtain wealth quintiles from 1 (lowest) to 5 (highest). Educational attainment was classified according to the Mexican educational system by categories: 5 years of schooling or fewer, 6 – 8 years, 9 – 11 years, or 12 years or more. Fertility was grouped categorically into 0 children, 1 – 2, 3 – 4, or 5 or more per woman. Age and the presence of a chronic condition (cancer, diabetes, or hypertension) were added as potential confounders.

**TABLE 1.** Distribution of participants, by social stratifier, Tijuana, 2014^a^

Variable^b^	Unweighted n	Unweighted %	Weighted %	95% CI
**Socioeconomic status (quintile)**				
Q1 (lower)	479	20.4	19.0	15 7 – 22 3
Q2	471	20.1	19.5	16.1 – 22.9
Q3	416	17.7	18.5	14.8 – 22.3
Q4	441	18.8	19.3	16.3 – 22.3
Q5 (higher)	468	20.0	20.7	15.8 – 25.5
Missing	70	3.0	2.9	2.2 – 3.7
**Educational attainment**				
≤5 years	580	24.7	23.9	20.5 – 27.3
6 to 8 years	718	30.6	30.4	25.4 – 35.4
9 to 11 years	631	26.9	27.2	23.7 – 30.8
12+ years	302	12.9	13.6	10.2 – 17.1
Missing	114	4.9	4.9	3.1 – 6.7
**Fertility**				
5+ children	191	8.1	7.8	6.3 – 9.2
3-4 children	668	28.5	28.6	24.3 – 32.9
0 children	482	20.6	20.4	17.5 – 23.3
1-2 children	989	42.2	42.5	37.2 – 47.8
Missing	15	0.6	0.7	0.3 – 1.2

***Source:*** Prepared by the authors from the study data.

a Unweighted n= 2 345, weighted = 491 084.

b Social stratifiers ordered from more to less disadvantaged

CI: Confidence interval

### Data analysis

A descriptive analysis of the prevalence of CSDS at each level of the social stratifiers was conducted, and inequality was measured with standard gap and gradient metrics: absolute and relative differences, the slope index of inequality (SII) and the concentration index (CIx). Unlike gap metrics, gradient metrics like SII and CIx allow for evaluations of inequalities across the full social spectrum. These inequality metrics were computed according to the WHO/PAHO guidelines (29).

The association between social stratifiers and the prevalence of CSDS was evaluated with multivariable logistic regression models. To assess intersectionality, two approaches were followed. First, terms for the interactions between social stratifiers were added to the regression model. As none of the terms had a *P* < 0.05, the final model included only main effects. Second, the prevalence of CSDS was calculated for participants at the intersections of the more disadvantaged categories, with results represented in a Venn diagram. RStudio (30) was used to conduct all analyses and produce the graphs.

### Ethics

Participants signed a letter of informed consent before responding to the survey questionnaire. To protect confidentiality, files containing personal data were kept in a secure location and accessed only by the researchers. The study was approved by the ethics committee of *El Colegio de la Frontera Norte* (#017-23-10-11), Tijuana, Mexico.

## RESULTS

There were 2 345 participants in the sample, representing a population of 491 084 women living in Tijuana in 2014. The mean age of participants was 37.0 years (95% CI: 36.5 – 37.4). The distribution by social stratifier appears in Table 1. Most participants had 6 – 8 years of education and 1 – 2 children.

### Social inequalities in CSDS distribution

**FIGURE 1.**
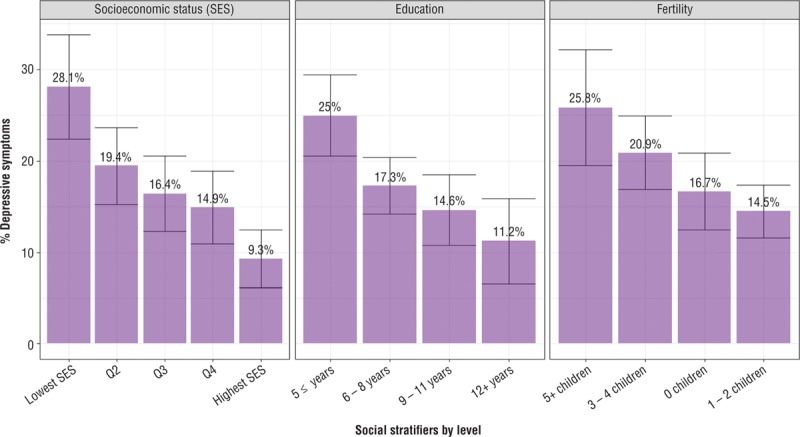
Inequality in depressive symptoms for social stratifiers among women in Tijuana, 2014

**FIGURE 2.**
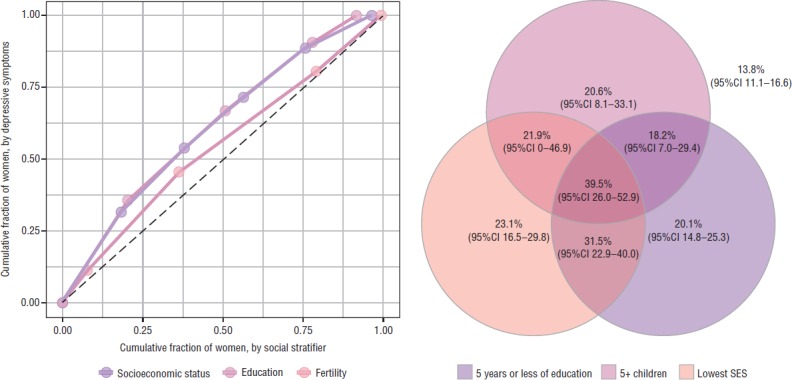
(A) Concentration curve and indexes of depressive symptoms among women in Tijuana, 2014. (B) Depressive symptoms among women of the most disadvantaged socioeconomic status, educational attainment and fertility levels, Tijuana, 2014

Overall prevalence of CSDS was 17.7% (95% CI: 15.1 – 21.0). The prevalence was higher among women with the lowest SES and educational levels, and among those with the highest and lowest fertility (Table 2 and Figure 1). The SII value was -21.9 (95% CI: -22.4 to -21.5) for SES, -17.0 (95% CI: -17.3 to -16.8) for educational attainment, and -11.2 (95% CI: -12.4 to -9.9) for fertility, reflecting the excess prevalence of CSDS among the more disadvantaged groups. Similarly, the concentration curves showed CSDS to be disproportionately concentrated in those groups (Figure 2A). The complete set of measures describing social inequality in the prevalence of CSDS is shown in Table 3.

### Intersectionality of social disparities in CSDS prevalence

None of the interaction terms between social stratifiers in the logistic regression were significant (results not shown). However, as shown in the Venn diagram (Figure 2B), women at the intersection of lowest-SES (1^st^ quintile of the wealth index), least-schooling (5 or fewer years of schooling), and higher-fertility (5 or more children) had a significantly higher prevalence of CSDS (39.5%; 95%CI: 26.0 – 52.9) as compared to women exhibiting none of these social disadvantages (13.8%; 95%CI: 11.1 – 16.6).

## DISCUSSION

The results of this study show profound inequalities in CSDS prevalence along socioeconomic and educational gradients among women in Tijuana. These findings concur with prior literature (13, 18, 19), including a previous study in the same city (24) that found a significant relationship between CSDS and SES and other social determinants in Latin America. As for fertility, a non-linear association was observed, so that participants with either less or more than 1 – 2 children had higher odds of CSDS. These participants were more likely to be single and to be students, so it is possible that their depression was related to life course stage. While adjusting by age partially accounted for this, the topic is worth exploring in future analysis.

**TABLE 2.** Prevalence and associations of clinically significant depressive symptoms, Tijuana, 2014^a^

Variable^b^	% depressive symptoms	95% CI	Adjusted OR^c^	95% CI	p-value
**Socioeconomic status (quintile)**					
Q1 (lower)	28.1	22.4 – 33.8	2.7	1.7 – 4.3	0.000
Q2	19.4	15.2 – 23.7	1.7	1.1 – 2.7	0.020
Q3	16.4	12.3 – 20.5	1.5	0.9 – 2.6	0.103
Q4	14.9	10.9 – 18.9	1.5	1.0 – 2.3	0.086
Q5 (higher)	9.3	6.1 – 12.4	Reference	Reference	Reference
**Educational attainment**					
≤5 years	25.0	20.5 – 29.4	1.8	1.0 – 3.0	0.046
6 to 8 years	17.3	14.2 – 20.3	1.1	0.7 – 1.9	0.606
9 to 11 years	14.6	10.7 – 18.5	1.2	0.7 – 1.9	0.508
12+ years	11.2	6.6 – 15.9	Reference	Reference	Reference
**Fertility**					
5+ children	25.8	19.5 – 32.2	1.7	1.0 – 2.8	0.046
3-4 children	20.9	16.8 – 24.9	1.5	1.0 – 2.0	0.019
0 children	16.7	12.5 – 20.8	1.4	1.0 – 2.0	0.097
1-2 children	14.5	11.6 – 17.4	Reference	Reference	Reference

***Source:*** Prepared by the authors from the study data.

a Unweighted n = 2 345, weighted = 491 084.

b Social stratifiers ordered from more to less disadvantaged

c Adjusted by socioeconomic status, educational attainment, fertility, age, age squared, and presence of chronic condition.

OR: odds ratio; CI: confidence interval.

Employing an intersectionality lens, this study considered that women may be situated in social contexts where multiple disadvantages cluster together. Instead of seeking to separate the effects of each social determinant, the concept of intersectionality invites researchers to account for the effects of these “clusters of disadvantage” (31). The prevalence of CSDS among women at the intersection of three vulnerable conditions was higher than the prevalence among women with only one, although the difference was not statistically significant in all cases. However, intersection terms in the regression models were not significant. Lack of significance could be explained by small sample sizes in some combinations of stratifiers. By depicting the situation of the more disadvantaged categories in each stratifier, the Venn diagram highlights inequities in the distribution of CSDS that could be missed by the regression method.

**TABLE 3.** Metrics of inequality of clinically significant depressive symptoms, Tijuana, 2014

Inequality metric^a^(disadvantaged/advantages)	Simple inequality metrics				Complex inequality metrics			
	Absolute gap	95%CI^b^	Relative gap	95% CI	SII^b^	95%CI^b^	CIx^b^	95% CI^b^
**Socioeconomic status**							
Quintile 1 / Quintile 5	18.8	16.2 – 21.4	3.1	2.7 – 3.7	-21.9	-22.3 – -21.6	0.16	0.14 – 0.19
**Educational attainment**							
≤5 years / 12+ years	13.7	13.5 – 14.0	2.3	1.8 – 3.1	-17.0	-17.2 – -16.8	0.07	0.05 – 0.11
**Fertility**							
5+ children / 1-2 children	11.3	7.8 – 14.8	1.8	1.7 – 1.9	-11.2	-12.3 – -10.1	0.08	0.07 – 0.08

***Source:*** Prepared by the authors from the study data.

a Social stratifiers ordered from more to less disadvantaged.

b SII: slope of inequality index; CIx: concentration index of health inequality; CI: confidence interval

A strength of this study was that it drew on representative results of the population of women in a border city in Mexico, with probabilistic methods and a robust sampling frame for three very relevant social stratifiers. Likewise, the measurement of health with respect to social stratifiers is a key aspect of prioritizing health equity and the SDH. Our assessment of gaps and other indices of inequality highlights a situation of profound inequalities for women, with fewer socioeconomic, schooling, and family planning resources at the extreme ends and throughout the social gradient. Few prior studies have explored the importance of the SDH in driving disparities in CSDS distribution, especially in Latin American countries. Furthermore, our assessment of the complex interactions between social stratifiers, such as SES, education, and fertility, provides unique information to better understand and improve women’s mental health in the Region of the Americas.

**Limitations.** A limitation of this study was the impossibility of including other, possibly important, social stratifiers such as ethnicity and migration. Preliminary versions of this analysis included stratifiers such as speaking an indigenous language, migration status, and the number of books in the home. Ethnicity is an important social determinant for Latin American populations; however, the only indicator available in the database was self-reported speaking an indigenous language, which was extremely rare in the sample (< 2%), and thus, the analysis lacked analytic power for this variable. Migration was not included as a social stratifier because it was not significantly associated with CSDS. In a city such as Tijuana, largely composed of migrants, many of whom are quite socioeconomically successful, it may be difficult to measure the specific circumstances in which migratory status conveys social disadvantage. Still, migrant status remains an important topic for further research. Although the number of books in a home is a strong social stratifier, the data were collected in broad categories that made analysis difficult. Moreover, we considered educational attainment to be a stronger social stratifier. The study also lacked data on social support and other psychosocial aspects (32), as well as on biological factors. Another potential limitation is the robustness and validity of the 10-item CES-D scale, but its psychometric properties have been proven satisfactory when compared with the original 20-item scale in several populations (5).

## Conclusions

This study made evident the social inequalities in CSDS and showed that disadvantages along multiple axes are associated with depressive symptoms among women in a large Latin American city. To address adverse social determinants, efforts to improve the mental health of women in the Region should include policies and interventions that are intersectional.

### Author contributions.

IB conceived and conducted the survey study. ACV and IB conceived the idea for this analysis and co-wrote the paper. ACV and OJM analyzed the data. All authors interpreted the results, and reviewed and approved the final version of the paper.

### Funding.

The data employed in this analysis were collected as part of a study funded by *the Consejo Nacional de Ciencia y Tecnología* of Mexico (Grant N°. CB-2010-153536). The funders had no role in the study design, data collection or analysis, decision to publish, or preparation of the manuscript.

### Disclaimer.

Authors hold sole responsibility for the views expressed in the manuscript, which may not necessarily reflect the opinion or policy of the *RPSP/PAJPH* and/or PAHO.
